# Prognostic Impact of Different Definitions of White-Coat Hypertension

**DOI:** 10.1093/ajh/hpaf136

**Published:** 2025-07-23

**Authors:** Paolo Verdecchia, Stefano Coiro, Claudia Bartolini, Adolfo Aita, Claudia Borgioni, Salvatore Repaci, Chiara Dembech, Massimo Guerrieri, Nicola Sacchi, Sergio Bistoni, Mario Trottini, Fabio Angeli

**Affiliations:** Associazione Umbra Cuore e Ipertensione and Division of Cardiology, Hospital S. Maria della Misericordia, Perugia, Italy; Division of Cardiology, Hospital S. Maria della Misericordia, Perugia, Italy; Division of Cardiology, Hospital S. Maria della Misericordia, Perugia, Italy; Departimento di Medicina,Unità Sanitaria Locale’Umbria 1’, Perugia, Italy; Departimento di Medicina,Unità Sanitaria Locale’Umbria 1’, Perugia, Italy; Departimento di Medicina,Unità Sanitaria Locale’Umbria 1’, Perugia, Italy; Departimento di Medicina, Ospedale di Castiglione del Lago, Perugia, Italy; Departimento di Medicina, Ospedale di Castiglione del Lago, Perugia, Italy; Departimento di Medicina, Ospedale di Castiglione del Lago, Perugia, Italy; Departimento di Medicina, Ospedale di Assisi, Perugia, Italy; Departimento di Medicina, Ospedale di Assisi, Perugia, Italy; Department of Medicine and Technological Innovation (DiMIT), University of Insubria, Varese, Italy; Department of Medicine and Cardiopulmonary Rehabilitation, Maugeri Care and Research Institute, IRCCS Tradate, Tradate, Italy

**Keywords:** ambulatory blood pressure monitoring, blood pressure, cardiovascular death, heart failure, hypertension, myocardial infarction, stroke, white-coat hypertension

## Abstract

**BACKGROUND:**

Different definitions of white-coat hypertension (WCH) may explain its variable outcome across studies.

**METHODS:**

In an Italian study started in 1986, we followed 3,153 people with (office blood pressure (BP) >=140/90 mmHg) and 457 without office hypertension for a mean of 10.4 years. None had previous cardiovascular disease. All underwent 24-h ambulatory BP (ABP) monitoring. We defined white-coat hypertension (WCH) as an average 24-h ABP < 130/80 mmHg or <125/75 mmHg. The primary outcome was a composite of major adverse cardiovascular events (MACE) and all-cause mortality.

**RESULTS:**

Baseline office BP was 156/97 mmHg in people with and 127/81 mmHg without hypertension. At follow-up, MACE events were 344 and 23, and all-cause deaths were 318 and 24 in people with and without hypertension, respectively. Compared to normotensive group, MACE risk was not higher in people with WCH and 24-h ABP < 125/75 mmHg (hazard ratio (HR), 0.94; 95% confidence interval (CI), 0.42–2.10). Compared to normotensive group, MACE risk was higher in people with WCH and 24-h ABP < 130/80 mmHg (HR: 1.79; 95% CI, 1.07–2.29). All-cause death did not differ between the normotensive group and people with WCH and 24-h ABP < 125/75 mmHg (HR 1.37; 95% CI, 0.68–2.73), but it was higher than in the normotensive group when WCH was defined by a 24-h ABP < 130/80 mmHg (HR 1.82; 95% CI, 1.55–3.58).

**CONCLUSIONS:**

WCH defined by an average 24-h ABP < 125/75 mmHg identifies people at low risk of MACE and death in the long term. Even modestly above these threshold values, the risk associated with WCH increases.

## Graphical Abstract

**Figure:**
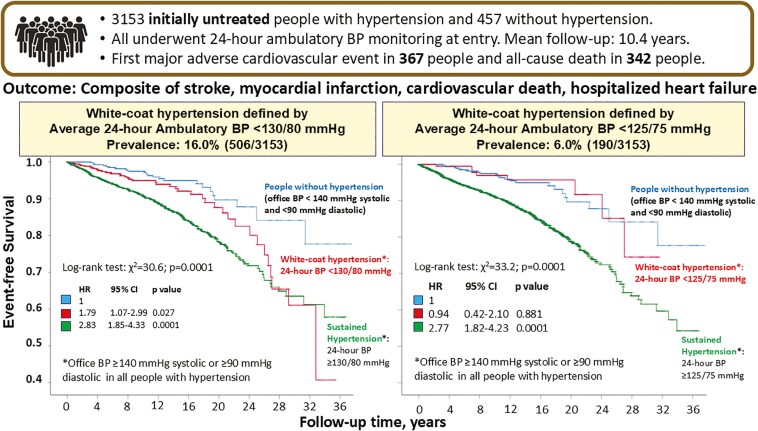
Prognostic impact of different guideline-suggested definitions of white-coat hypertension.

More than three decades after the first outcome studies,^1–3^ the prognostic impact of white-coat hypertension (WCH) remains controversial.^4–10^ Whereas some outcome studies showed a higher risk of major adverse cardiovascular events (MACE) and mortality in hypertensive people with WCH compared with clinically normotensive people,^5,11^ other studies failed to detect outcome differences between the two groups.^4,5,12^ This uncertainty is reflected in the results of some meta-analyses published on the subject.^13–16^

A shared definition of WCH would be important because several factors could make this phenotype heterogeneous.^17^ In some studies, but not in all,^18^ WCH was associated with a lesser risk of outcome events in treated than in untreated people.^14,15^ Potential reasons for the different prognostic impact of WCH across studies could be related to differences in some determinants of WCH, including age, sex, cigarette smoking, and organ damage.^4,7^ Also the duration of follow-up could play a role. The incidence of stroke among people with WCH may not increase in the short term, but it could increase in the long term.^19^ Also important is the cardiovascular risk profile in the normotensive control population, which in some studies included,^18^ while in others excluded,^11^ people with masked hypertension (MH). The removal of high-risk individuals with MH from the normotensive control group may unmask an increased cardiovascular risk profile in people with WCH.^20,21^

Another possible explanation of the discrepancy among available outcome studies on WCH may be the definition of WCH itself. Using 24-h ambulatory blood pressure (ABP) to define WCH in people with office diagnosis of hypertension and adopting the threshold values proposed by the North American 2017 AHA/ACC guidelines,^22^ we should define WCH by 24-h ABP values < 125/75 mmHg. Conversely, if we use the thresholds proposed by the European ESH^23^ and ESC^24^ guidelines and the ISH^25^ guidelines, we should define WCH using 24-h ABP values < 130/80 mmHg.

It is unclear whether these apparently modest differences in the diagnosis of WCH translate into outcome differences. In view of the impact of this issue in clinical practice, we investigated for the first time the prevalence and the long-term prognostic impact of WCH using the different ABP thresholds described above.^22–25^ Both the hypertensive and the normotensive control groups were composed of initially untreated people without previous cardiovascular disease.

## METHODS

This article adheres to the American Heart Association’ implementation of the Transparency and Openness Promotion Guidelines. Because of the sensitive nature of the data collected for this study, requests to access the dataset from qualified researchers trained in human subject confidentiality protocols may be sent to the corresponding author.

The Progetto Ipertensione Umbria Monitoraggio Ambulatoriale (PIUMA) study was initiated in Umbria, Central Italy, in June 1986. The last follow-up contact was made in April 2025. PIUMA is a prospective observational registry in initially untreated hypertensive people, with long-term follow-up. The registry was approved by the Local Ethics Committee of the Italian National Health Service (Protocol 2424/III and 4774/24). All subjects gave informed consent to participate. Details of the study have been previously published.^1,26,27^

In people with hypertension, entry criteria included an office BP ≥ 140 mm Hg systolic or ≥ 90 mm Hg diastolic in at least three visits over the last weeks and the absence of secondary causes of hypertension, previous cardiovascular disease, cancer, or other life-threatening conditions. At entry, all hypertensive people were not receiving antihypertensive drugs for at least 4 weeks. A cohort of 457 healthy normotensive people, members of the hospital staff, medical students, or training fellows without previous cardiovascular disease, were included as a control group. To be eligible, their office BP had to be less than 140 mmHg systolic and 90 mmHg diastolic for at least three visits over a 3-week period in the absence of any treatment.

Office BP was measured by a physician using a mercury sphygmomanometer with subjects seated and relaxed for at least 10 minutes. Cuff size was adjusted for arm circumference. Three readings were averaged for analysis. Systolic and diastolic BPs were determined according to Korotkoff phases I and V.

We recorded 12-lead electrocardiography (ECG) at 25 mm/s and 1 mV/cm calibration. We excluded people with complete right or left bundle branch block, previous myocardial infarction (MI), Wolff-Parkinson-White syndrome, or atrial fibrillation at baseline. None of the subjects were receiving digitalis. We made diagnosis of left ventricular (LV) hypertrophy at ECG using a score developed in our laboratory,^28^ which requires the positive answer to ≥1 of the following two criteria: typical strain pattern in leads V_4_, V_5_, V_4_, D1, or aVL, or product of the Cornell voltage (R wave height in lead aVL plus S wave depth in lead V_3_) by body mass index (BMI) > 604 mm ∙ kg/m^2^. In a previous study, we found that correction of Cornell voltage by BMI as a marker of obesity improves the performance of traditional electrocardiography for diagnosis of LV hypertrophy in hypertensive patients.^28^

We made diagnosis of diabetes by fasting plasma glucose ≥ 7.0 mmol/L (126 mg/dl) or current antidiabetic therapy.^29^

### Ambulatory blood pressure

All people with and without office hypertension underwent 24-h ABP monitoring. We used oscillometric devices (SpaceLabs 520019, 9020220, and 9020721, SpaceLabs, Redmond, Washington, USA). The frequency of measurements was set to one every 15 minutes throughout the 24-h period. We defined daytime and nighttime ABP using arbitrarily defined narrow fixed clock intervals (from 10 am to 8 pm for day and from midnight to 6 am for night), which excludes the morning and evening transition periods during which a variable proportion of subjects are actually awake or asleep and may provide an accurate estimate of true BP values during sleep and wakefulness.^30^ We excluded shift workers from the study. We investigated the reproducibility of ABP measurements in our laboratory in a previous study in which a random sample of untreated hypertensive people enrolled in the PIUMA registry underwent repeated 24-h BP monitoring within 3–5 days.^31^ The between-session coefficient of variability (standard deviation (SD) of the mean of the paired differences between two sessions divided by the mean of all paired means) was 5.9%/6.3% for daytime BP and 6.1%/6.3% for nighttime BP.^31^

We defined WCH using the average 24-h ABP, with the normal values suggested by AHA/ACC (i.e., < 125/75 mmHg),^22^ and by ESH,^23^ ESC,^24^ and ISH^25^ (i.e., <130/80 mmHg).

#### Follow-up

Patients were followed by their primary care physicians in collaboration with our hospital staff. Treatment was individualized with lifestyle and pharmacological measures. It is difficult to predict to what extent the results of the 24-h ABP monitoring available to the physicians influenced the management of individual subjects. The most commonly used antihypertensive medications were thiazide diuretics, β-blockers, angiotensin-converting enzyme inhibitors, angiotensin II (AT-II) antagonists, calcium channel blockers, and α1-blockers, alone or in combination. We scheduled regular contacts with primary care physicians, telephone interviews, and clinical visits with patients to assess vital status and the occurrence of events.

#### Assessment of endpoints

We reviewed in conference the medical records and other source documents of patients who died or developed a first cardiovascular event and adjudicated each event. We analyzed a composite pool of first cardiovascular events and all-cause mortality as end points. Cardiovascular events included fatal or nonfatal MI, fatal or nonfatal stroke, congestive heart failure requiring hospitalization, sudden cardiac death, and other cardiovascular death. Soft endpoints, including transient ischemic attack, angina, non-MI revascularization, peripheral arterial disease, and dialysis, were excluded as terminating events. The standard international criteria for the diagnosis of outcome events in the PIUMA trial have been previously described.^32^

#### Data analysis

Analysis was performed with SPSS Statistics release 18 (IBM SPSS Statistics) and Stata release 16.0 (College Station, Texas 77845 USA). Parametric data are presented as mean ±  SD. Survival curves were estimated using the Kaplan–Meier product-limit method^33^ and compared using the Mantel test (log-rank).^34^ The rates of events are reported along with their 95% confidence intervals (CI). The effect of prognostic factors on survival was evaluated using Cox semiparametric regression models.^35^ The hazard ratio (HR) and its 95% CI linked to 24-h ABP was tested after adjustment for the following variables: age, sex (female, male), diabetes mellitus (no, yes), smoking status (current smoker, never smoker), drug treatment at follow-up (no, yes), serum uric acid (1 mmol/l), LV hypertrophy at ECG (no, yes) and low-density lipoprotein cholesterol (1 mmol/l). Since all-cause mortality could act as a competing risk factor on MACE because individuals who die from other causes are no longer at risk for MACE, we analyzed MACE after allowance for all-cause death as a competing risk factor. We used the Fine and Gray method,^36^ implemented in Stata through the “stcrreg” command. Two-sided *P*-values ≤0.05 were considered statistically significant.

## RESULTS

**Figure 1.**
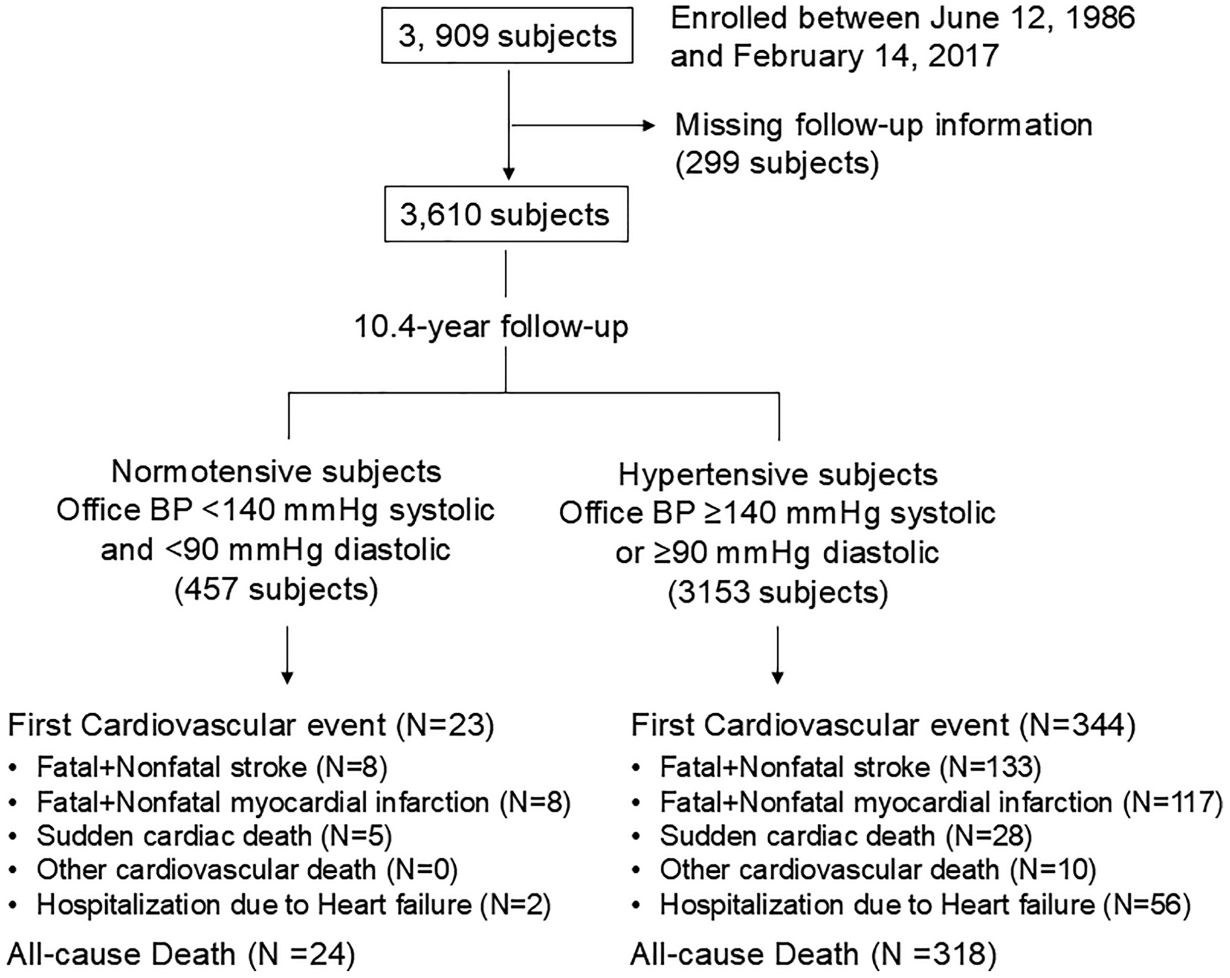
Flow diagram of the study.

Out of 3,909 people who entered the study, we obtained data from 3,610 people, who were followed for a mean of 10.4 years (**Figure 1**). Mean office BP was 156/97 mmHg in the hypertensive group and 127/81 mmHg in the normotensive control group.

Prevalence of WCH was 16.0% using the definition suggested by the 2023 ESH, 2024 ESC, and 2020 ISH Guidelines (average 24-h ABP < 130/80 mmHg), and 6.0% using the definition suggested by the 2017 AHA/ACC BP Guidelines (average 24-h ABP < 125/75 mmHg). Data are graphically reported in **Supplementary Figure S1**. **Table 1** shows the main features of the population according to the two different definitions of WCH.

**Table 1.** Main features of the population

	All	Normotensive Group	Hypertensive Group: 24-h ABP <130/80 mmHg	Hypertensive Group: 24-h ABP >=130/80 mmHg	Hypertensive Group: 24-h ABP <125/75 mmHg	Hypertensive Group: 24-h ABP >=125/75 mmHg
Number	3,610	457	506	2,647	190	2,963
Age (years)	49.9 (12)	44.7 (13)	50.0 (12)^*^	50.7 (12)^*^	52.1 (13)^*^	50.6 (12)
Sex (% women)	1629 (45.1)	207 (45.3)	314 (62.1)^*^	1108 (41.9)	134 (70.5)^*^	1288 (43.5)
Body mass index (kg/m^2^)	26.8 (3.8)	26.2 (3.6)	26.9 (4.0)^*^	26.9 (3.9)^*^	27.1 (4.3)	26.8 (3.8)
Office systolic BP (mmHg)	152.7 (20)	127.2 (8)	147.2 (14)^*^	158.2 (19)^*^	147.7 (13)^*^	157.0 (19)
Office diastolic BP (mmHg)	95.1 (11)	81.1 (6)	91.4 (8)^*^	98.3 (10)^*^	98.1 (10)^*^	90.7 (8)
24-h systolic BP (mmHg)	134.9 (15)	120.5 (9)	119.9 (6)	140.3 (13)^*^	115.7 (6)^*^	138.4 (14)
24-h diastolic BP (mmHg)	85.5 (10)	76.6 (7)	74.0 (4)	89.2 (9)^*^	70.0 (4)^*^	87.9 (9)
Daytime systolic BP (mmHg)	141.2 (15)	126.3 (9)	126.6(8)^*^	146.6 (13)	122.1 (7)^*^	144.7 (14)
Daytime diastolic BP (mmHg)	91.4 (11)	82.1 (8)	80.4 (6)^*^	95.2 (9)^*^	76.2 (5)	93.9 (10)
Nighttime systolic BP (mmHg)	123.0 (17)	109.3 (10)	108.4 (9)	128.1 (16)^*^	104.6 (8)^*^	126.2 (16)
Nighttime diastolic BP (mmHg)	74.8 (11)	66.6 (8)	63.7 (6)^*^	78.4 (10)^*^	60.1 (5)^*^	77.0 (10)
Cigarette smoking (%)	898 (24.9)	138 (30.3)	92 (18.2)^*^	668 (25.2)^*^	29 (15.3)^*^	731 (24.7)^*^
Diabetes (%)	290 (8.9)	33 (8.5)	35 (6.9)^*^	221 (8.4)	12 (6.3)^*^	244 (8.2)
Left ventricular hypertrophy (%)	550 (17.6)	27 (7.1)	36 (7.9)	487 (21.3)^*^	9 (5.1)	514 (20.1)^*^
Total cholesterol (mmol/l)	5.56 (1.09)	5.32 (1.04)	5.62 (1.17)^*^	5.59 (1.08)^*^	5.62 (1.17)^*^	5.59 (1.09)
HDL cholesterol (mmol/l)	1.28 (0.32)	1.27 (0.32)	1.31 (0.29)	1.27 (0.33)	1.30 (0.28)	1.28 (0.32)
LDL cholesterol (mmol/l)	3.58 (0.96)	3.42 (0.92)	3.67 (1.01)^*^	3.58 (0.95)^**^	3.66 (0.94)	3.59 (0.95)
Glucose (mmol/l)	5.56 (1.27)	5.48 (1.19)	5.50 (1.10)	5.59 (1.32)	5.48 (1.18)	5.58 (1.29)
Creatinine (mmol/l)	86.2 (20)	85.2 (16)	82.2 (15)	87.2 (21)	80.2 (14)	86.8 (20)
Uric acid (mmol/l)	284.2 (80)	273.2 (80)	268.4 (74)	288.8 (85)^*^	261.3 (74)	287.1 (84)
Potassium (mEq/l)	4.21 (0.38)	4.23 (0.36)	4.24 (0.36)	4.20 (0.39)	4.24 (0.35)	4.20 (0.39)

Values expressed as mean (± SD when appropriate) or proportion. Abbreviations: BP, blood pressure; HDL, high-density lipoprotein; LDL, low-density lipoprotein.

^*^ *P* < 0.01 vs. normotensive group.

^**^ *P* < 0.05 vs. normotensive group.

At the last follow-up visit, the proportion of people currently treated with antihypertensive drugs was significantly lower in people with WCH than in that with sustained hypertension regardless of the definition of WCH (all *P* < 0.01) (**Supplementary Figure S2**).

### Analysis of MACE

As shown in **Figure 1**, during follow-up, there were 23 MACE events in people without hypertension (0.42 [95% CI, 0.32–0.51] per 100 patient-years) and 344 MACE events in people with hypertension (1.07 [95% CI, 1.02–1.12] per 100 patient-years). All-cause deaths were 24 in the normotensive group and 318 in the hypertensive group.

**Figure 2.**
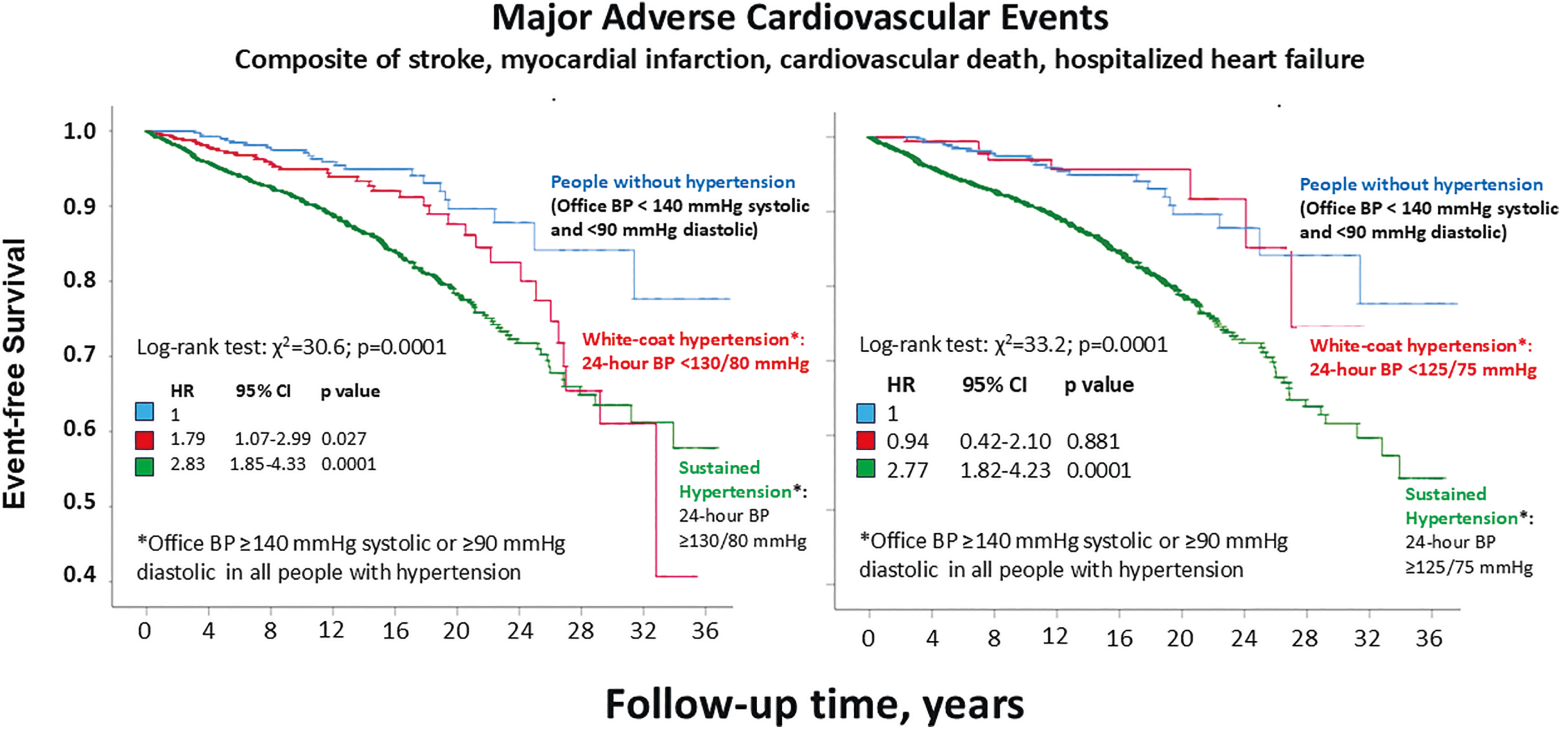
Major adverse cardiovascular events.

Compared to the normotensive group, the MACE rate (**Figure 2**, left panel) was higher in people with WCH defined by an average 24-h ABP < 130/80 mmHg (0.42 [95% CI, 0.32.0.51] vs. 0.70 [95% CI, 0.61–0.79] events per 100 patient-years; HR 1.79; 95% CI, 1.07–2.99; *P* = 0.027) and higher in those with sustained hypertension (1.04 [95% CI, 1.00–1.08] events per 100 patient-years; HR 2.83 95% CI, 1.85–4.33; *P* = 0.0001).

Conversely, compared to the people without hypertension, the incidence of MACE events (**Figure 2**, right panel) did not differ in people with WCH defined by an average 24-h ABP < 125/75 mmHg (0.42 [95% CI, 0.32–0.51] vs. 0.37 [95% CI 0.28–0.47] events per 100 patient-years; HR 0.94; 95% CI, 0.42–2.10; *P* = 0.881), while it increased in people with sustained hypertension (1.02 [95% CI, 0.99–1.05] events per 100 patient-years; HR 2.77; 95% CI, 1.82–4.23; *P* = 0.0001).

The results of Cox multivariable analysis for MACE after competing risk adjustment for all-cause death are reported in **Supplementary Table S1**. Analysis of all-cause death is reported in **Supplementary Tables S1** and **S2**.

**Supplementary Figure S3** reports the risk of MACE in people with average 24-h ABP < 125/75 mmHg, 125–129/75–79 mmHg, and >= 130/80 mmHg for defining WCH. Compared to normotensive people, MACE rate was not increased in the subgroup with 24-h ABP < 125/75 mmHg (HR 0.92; 95% CI, 0.42.2.10; *P* = 0.876). However, MACE rate was higher in the subgroup with 24-h ABP 125–129/75–79 mmHg (HR 2.31; 95% CI, 1.35–3.94; *P* = 0.002) and even higher in the subgroup with sustained hypertension.

### Analysis of all-cause death

**Figure 3.**
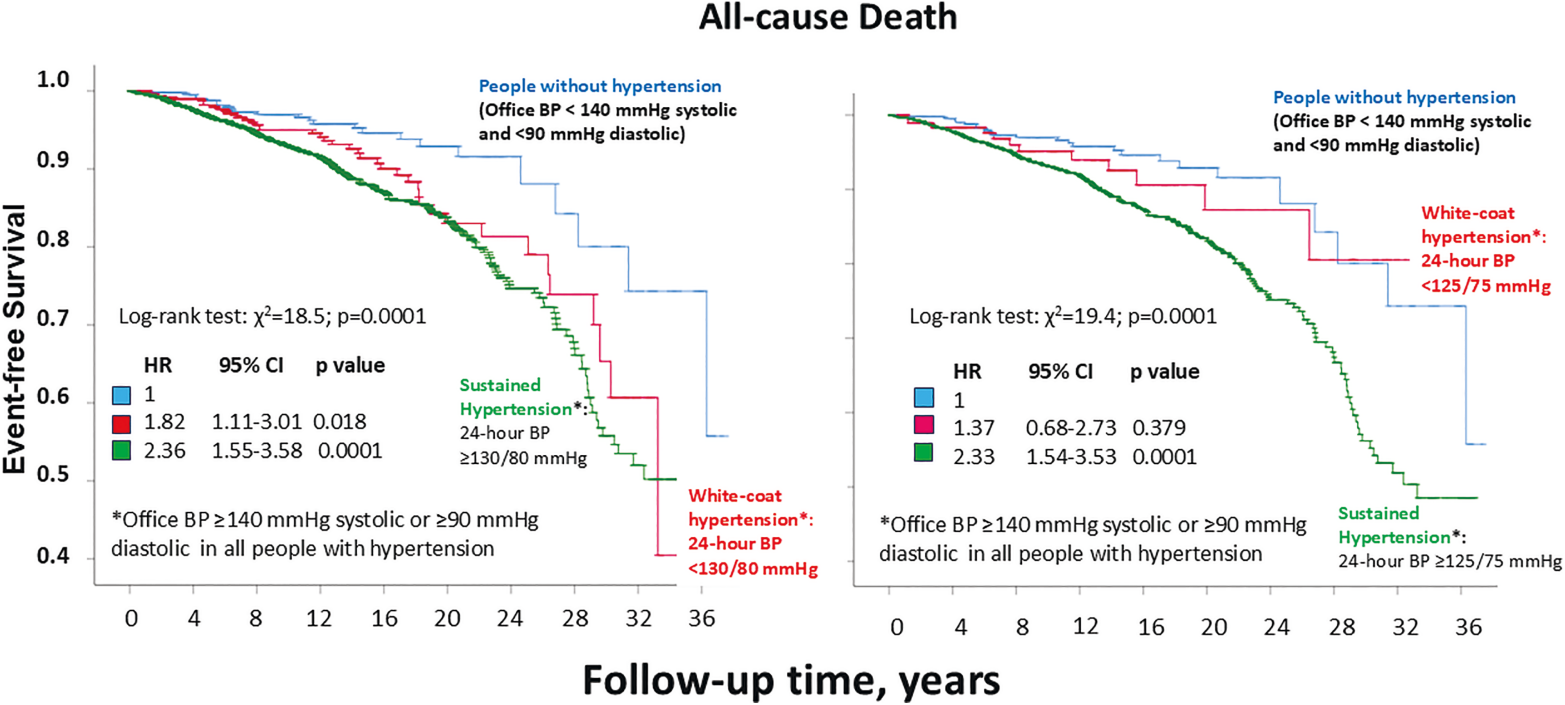
All-cause death.

Results for all-cause death are reported in **Figure 3**. Compared to normotensive people, incidence of all-cause death (left panel) was higher in people with WCH defined by an average 24-h ABP < 130/80 mmHg (0.42 [95% CI, 0.32–0.52] vs. 0.75 [95% CI, 0.67–0.83] events per 100 patient-years; HR 1.82; 95% CI, 1.11–3.01; *P* = 0.018) and also higher in people with sustained hypertension (0.94 [95% CI, 0.89–0.99] events per 100 patient-years; HR 2.36; 95% CI, 1.55–3.58; *P* = 0.0001). In contrast, compared to normotensive people, the incidence of all-cause death (right panel) did not differ in people with WCH group defined by an average 24-h ABP < 125/75 mmHg (0.42 [95% CI, 0.32–0.52] vs. 0.56 [95% CI, 0.46–0.66] events per 100 patient-years; HR 1.37; 95% CI, 0.68–2.73; *P* = 0.379), and increased in those with sustained hypertension (0.93 [95% CI, 0.88–0.98] events per 100 patient-years; HR 2.33; 95% CI, 1.54–3.53; *P* = 0.0001).

## DISCUSSION

Compared to a less stringent definition of normal ABP (24-h ABP < 130/80 mmHg), a slightly tighter definition (24-h ABP < 125/75 mmHg) was associated with a significantly lower prevalence of WCH and a lower risk of MACE events and death. These findings were obtained in a cohort of people with untreated hypertension and no established cardiovascular disease. A previous study by Cuspidi et al in untreated hypertensive people^37^ found a 9.4% prevalence of WCH using the 125/75 mmHg definition^22^ and a 17.4% prevalence using the 130/80 mmHg definition,^38^ against 6.0% and 16%, respectively, in the present study. The prevalence of WCH did not differ remarkably between the study by Cuspidi^37^ and the present study.

The WCH group defined by 2017 AHA/ACC BP Guidelines^22^ was lower in size when compared to that defined by ESH,^23^ ESC,^24^ or ISH,^25^ but it showed definite markers of low cardiovascular risk. For example, the average 24-h ABP was lower than in the normotensive control group (115.7 mmHg vs. 120.5 mmHg, respectively; all *P* < 0.01). Also, the prevalence of diabetes mellitus was lower than in the normotensive control group (6.3% vs. 8.5%, *P* < 0.01). Conversely, these parameters did not show significant differences between the normotensive people and the WCH group defined using an average 24-h ABP < 130/80 mmHg.

These results should be considered in the context of meta-analyses and individual studies focused on the prognostic impact of WCH. In a meta-analysis of longitudinal studies that used different definitions of WCH, Pierdomenico and Cuccurullo did not find any significant differences in the risk of major cardiovascular events in people with WCH compared with the normotensive control group.^16^ In a subsequent meta-analysis of 14 studies, for a total of 4,806 patients with WCH and 13,538 clinically normotensive patients, WCH was associated with an increased risk of a composite pool of major cardiovascular events (4% vs. 6%, respectively), but not of all-cause death or stroke.^13^ The definition of WCH varied consistently, being based on a daytime ABP < 135/85 mmHg in most studies, and by self-measured home BP alone or pooled with ABP in other studies.^13^ In a subsequent larger meta-analysis of 23 studies, WCH was associated with a 38% higher risk of major cardiovascular events compared to the control group in initially untreated, but not in treated people.^15^ Again, the definition of WCH differed across the studies, being based on a daytime ABP < 135/85 mmHg in most studies, and by home BP in other studies.^15^ These findings were substantially confirmed in a larger meta-analysis of 27 studies, which showed a significantly higher risk of MACE, cardiovascular mortality, and all-cause mortality in the WCH group compared with the normotensive control group in untreated people.^14^ Again, WCH was defined by a daytime ABP < 135/85 mmHg in some studies, an average 24-h ABP < 130/80 mmHg in other studies and by self-measured home BP in other studies.^14^ The analysis found an increased risk of major cardiovascular events in the WCH group compared with the normotensive group in the totality of studies, but not in the subgroup of studies in which WCH was defined by mean 24-h ambulatory values < 130/80 mmHg, suggesting that different definitions of WCH could impact its prognostic value.^14^

### Limitations and strengths

A limitation of our study was the relatively small number of people and outcome events in the group without hypertension along with the rather remote start date of our study, which began in 1986. Another limitation is the lack of North Americans and the use of the 140/90 mmHg threshold to define office hypertension, which is not universally accepted as the standard.^22,39^ Strengths of the study are the long duration of follow-up and the absence of previous antihypertensive treatment at entry in all people, which make our findings applicable to initially untreated people with office hypertension. Another unique feature of the present study was the exclusion of individuals with established cardiovascular disease, a condition that could have increased cardiovascular risk in people with WCH.^20^ This is an important point because a previous MACE would in any case exclude people with WCH from a definition of low risk, regardless of the definition of this phenotype based on out-of-office BP. We defined the normotensive group based solely on office BP. Therefore, subjects with MH may have been present in the normotensive group.

Franklin *et al*. showed that the inclusion of treated hypertensive people at baseline ABP monitoring doubled the risk of major cardiovascular events compared with a normotensive control group.^18^ The authors hypothesized that antihypertensive treatment at baseline could somewhat mimic a WCH state while failing to reverse the previous damage caused by the hypertensive state.^18,21^ In our study, a variable proportion of people with WCH, ranging from 53.8% to 57.2% depending on the different definitions, were receiving antihypertensive drug treatment at follow-up. Therefore, the diagnosis of WCH at baseline was not a consistent reason to exclude these people from antihypertensive treatment at follow-up, although the proportion of treated people was lower than in the group with ambulatory hypertension.

To the best of our knowledge, the novel finding of this study is that even modest differences in the definition of WCH using 24-h ABP are associated with outcome differences in terms of MACE and all-cause death. The take-home message is that a more stringent definition of WC based on an average 24-h ABP < 125/75 mmHg should be preferred for the diagnosis of WCH in initially untreated hypertensive people encountered in clinical practice.

## Supplementary Material

hpaf136_suppl_Supplementary_Materials_1
