# Supplementary material for: Prognostic Impact of Different Definitions of White-Coat Hypertension
Source: Am J Hypertens. 2025 Jul 23;38(12):1043–50. doi: 10.1093/ajh/hpaf136 (PMC12620022; doi:10.1093/ajh/hpaf136)
Supplement: hpaf136_suppl_Supplementary_Materials_1 [file hpaf136_suppl_supplementary_materials_1.docx]

**Supplemental Data**

**Prognostic Impact of Different Definitions of White-Coat Hypertension**

**Paolo Verdecchia^1^, Stefano Coiro^2^, Claudia Bartolini^2^, Adolfo Aita^3^, Claudia Borgioni^3^, Salvatore Repaci^3,^ Chiara Dembech^4^, Massimo Guerrieri^4^, Nicola Sacchi^4^, Sergio Bistoni^5^, Mario Trottini^5^, Fabio Angeli^6^**

^1^ Associazione Umbra Cuore e Ipertensione and Division of Cardiology, Hospital S. Maria della Misericordia, Perugia, Italy;

^2^Division of Cardiology, Hospital S. Maria della Misericordia, Perugia, Italy

^3^Unità Sanitaria Locale ’Umbria 1’, Perugia, Italy

^4^Ospedale di Castiglione del Lago, Perugia, Italy

^5^Ospedale di Assisi, Perugia, Italy.

^6^ Department of Medicine and Technological Innovation (DiMIT), University of Insubria, Varese and Department of Medicine and Cardiopulmonary Rehabilitation, Maugeri Care and Research Institute, IRCCS Tradate, Italy;

Table/Figure Page

**Table S1**.

Major Adverse Cardiovascular Events. Multivariate analysis….………… 2

**Table S2**.

All-cause death. Multivariate analysis……………………………………. 3

**Figure S1**.

Prevalence of white-coat hypertension

using different definitions…………………………..……………….……. 4

**Figure S2**.

Proportion of subjects receiving antihypertensive drugs

at the last follow-up visit…………………………………………………. 5

**Figure S3**. Major adverse cardiovascular events in people with different

levels of average 24-hour ambulatory blood pressure used for definition

of white-coat hypertension……………………………………………….. 6

**Table S1**. Main features of the population according to the different definitions of white-coat hypertension.

____________________________________________________________________________________________________________________

Normotensive White-coat Ambulatory White-coat Ambulatory

Group Hypertension Hypertension Hypertension Hypertension

----------------------------------------------- --------------------------------------------------

24-hour ABP: 24-hour ABP: 24-hour ABP: 24-hour ABP:

<130/80 mmHg >=130/80 mmHg <125/75 mmHg >=125/75 mmHg

----------------------------------------------------------------------------------------------------------------------------------------------------------------------------------Number 457 506 2647 190 2963

Age (years) 44.7 (13) 50.9 (12) Ϯ 50.7 (12) Ϯ 52.1 (13) Ϯ 50.6 (12) Ϯ

Sex (% women) 207 (45) 314 (62) Ϯ 1108 (42) 134 (70) Ϯ 1288 (43)

Weight (kg) 74.7 (13) 73.6 (13) 76.0 (14) 73.3 (14) 75.8 (14)

Height (cm) 168.5 (9) 165.4 (9) 167.9 (9) Ϯ 164.2 (8) 167.7 (9) Ϯ

Body mass index (kg/m^2^) 26.2 (3.6) 26.9 (4.0) ǂ 26.9 (3.9) Ϯ 27.1 (4) Ϯ 26.8 (4) Ϯ

Office systolic BP (mmHg) 127.2 (8) 147.2 (14) Ϯ 158.2 (19) Ϯ 147.7 (13) Ϯ 157.0 (19) Ϯ

Office diastolic BP (mmHg) 81.0 (6) 91.4 (8) Ϯ 98.3 (10) Ϯ 90.1 (8) Ϯ 97.6 (10) Ϯ

24-hour systolic BP (mmHg) 120.5 (8) 119.9 (6) 140.3 (13) Ϯ 115.7 (6) Ϯ 138.4 (14) Ϯ

24-hour diastolic BP (mmHg) 76.6 (7) 74.0 (4) Ϯ 89.2 (9) Ϯ 70.0 (4) Ϯ 87.9 (9) Ϯ

Daytime systolic BP (mmHg) 126.3 (9) 126.6 (8) 146.6 (13) Ϯ 122.1 (7) Ϯ 144.7 (14) Ϯ

Daytime diastolic BP (mmHg) 82.1 (8) 80.4 (6) Ϯ 95.2 (9) Ϯ 76.2 (5) Ϯ 93.9 (10) Ϯ

Nighttime systolic BP (mmHg) 109.3 (10) 108.4 (9) 128.1 (16) Ϯ 104.6 (8) Ϯ 126.2 (16) Ϯ

Nighttime diastolic BP (mmHg) 66.6 (8) 63.7 (6) Ϯ 78.4 (11) Ϯ 60.1 (5) Ϯ 77.0 (10) Ϯ

Current cigarette smoking (%) 138 (30.3) 92 (18.2) Ϯ 668 (25.2) 29 (15.3) Ϯ 731 (24.7)

Diabetes (%) 33 (8.5) 35 (7.7) 222 (9.2) 12 (6.9) Ϯ 245 (9.1)

Left ventricular hypertrophy (%) 27 (7.1) 36 (7.9) 487 (21.3) Ϯ 9 (5.1) 514 (20.1) Ϯ

Total cholesterol (mmol/l) 5.24 (1.04) 5.62 (1.17) Ϯ 5.59 (1.08) Ϯ 5.62 (1.2) Ϯ 5.59 (1.1) Ϯ

HDL cholesterol (mmol/l) 1.27 (0.31) 1.31 (0.29) 1.27 (0.33) 1.30 (0.28) 1.28 (0.33)

LDL cholesterol (mmol/l) 3.42 (0.92) 3.67 (1.01) Ϯ 3.58 (0.95) Ϯ 3.66 (0.94) ǂ 3.59 (0.96) Ϯ

Glucose (mmol/l) 5.47 (1.19) 5.50 (1.10) 5.59 (1.32) ǂ 5.47 (1.18) 5.58 (1.29)

Creatinine (mmol/l) 85.1 (16) 82.2 (15) ǂ 87.2 (21) ǂ 80.2 (14) 86.8 (20) ǂ

Uric acid (mmol/l) 284.1 (83) 273.2 (80) 285.6 (83) Ϯ 261.3 (74) 287.1 (84) Ϯ

Potassium (mEq/l) 4.21 (0.38) 4.23 (0.36) 4.21 (0.39) 4.25 (0.35) 4.20 (0.39)

Values expressed as mean (± standard deviation when appropriate) or proportion. Abbreviations: ABP=ambulatory blood pressure; BP=blood pressure; HDL=high density lipoprotein; LDL=low density lipoprotein. Ϯ = p<0.01 vs normotensive group; ǂ = p<0.05 vs normotensive group.

**Table S2**. Major Adverse Cardiovascular Events. Multivariate analysis.

_________________________________________________________________________

Covariate Comparison Hazard ratio P

(95% CI) value

-----------------------------------------------------------------------------------------------------------------------

Average 24-hour Systolic BP 14 mmHg (1 SD) 1.43 (1.25-1.65) 0.0001

Age 11 years (1 SD) 1.97 (1.67-2.33) 0.0001

Sex 0=women; 1=men 1.67 (1.67-2.40) 0.005

Diabetes 0=no; 1=yes 2.27 (1.57-3.27) 0.0001

Cigarette smoking 0=no; 1=yes 2.22 (1.63-3.03) 0.0001

LDL cholesterol 0.96 mmol/l (1 SD) 1.21 (1.05-1.39) 0.008

Serum uric acid 84 mmol/l (1 SD) 1.32 (1.13-1.54) 0.0001

LV hypertrophy on ECG 0=no; 1=yes 1.50 (1.09-2.07) 0.012

Average Daytime Systolic BP 15 mmHg (1 SD) 1.39 (1.21-1.60) 0.0001

Age 11 years (1 SD) 2.04 (1.72-2.40) 0.0001

Sex 0=women; 1=men 1.71 (1.19-2.45) 0.004

Diabetes 0=no; 1=yes 2.32 (1.61-3.34) 0.0001

Cigarette smoking 0=no; 1=yes 2.24 (1.64-3.05) 0.0001

LDL cholesterol 0.96 mmol/l (1 SD) 1.20 (1.05-1.38) 0.009

Serum uric acid 84 mmol/l (1 SD) 1.33 (1.14-1.55) 0.0001

LV hypertrophy on ECG 0=no; 1=yes 1.58 (1.15-2.17) 0.005

Average Night-time Systolic BP 17 mmHg (1 SD) 1.55 (1.34-1.80) 0.0001

Age 11 years (1 SD) 1.88 (1.59-2.22) 0.0001

Sex 0=women; 1=men 1.74 (1.22-2.49) 0.001

Diabetes 0=no; 1=yes 2.18 (1.51-3.15) 0.0001

Cigarette smoking 0=no; 1=yes 2.29 (1.68-3.11) 0.0001

LDL cholesterol 0.96 mmol/l (1 SD) 1.21 (1.57-1.40) 0.007

Serum uric acid 84 mmol/l (1 SD) 1.31 (1.12-1.52) 0.001

LV hypertrophy on ECG 0=no; 1=yes 1.43 (1.04-1.98) 0.029

_________________________________________________________________________

Abbreviations: SD=Standard deviation; LDL=low density lipoprotein; ECG=electrocardiogram; BP=blood pressure.

**Table S3**. All-cause death. Multivariate analysis.

_________________________________________________________________________

Covariate Comparison Hazard ratio P

(95% CI) value

-----------------------------------------------------------------------------------------------------------------------

Average 24-hour Systolic BP 14 mmHg (1 SD) 1.32 (1.17-1.48) 0.0001

Age 11 years (1 SD) 3.15 (2.74-3.63) 0.0001

Sex 0=women; 1=men 1.61 (1.21-2.15) 0.001

Diabetes 0=no; 1=yes 1.78 (1.33-2.39) 0.0001

Cigarette smoking 0=no; 1=yes 1.69 (1.26-2.25) 0.0001

Serum uric acid 84 mmol/l (1 SD) 1.15 (1.01-1.31) 0.041

Average Daytime Systolic BP 14 mmHg (1 SD) 1.32 (1.17-1.50) 0.0001

Age 11 years (1 SD) 3.24 (2.82-3.73) 0.0001

Sex 0=women; 1=men 1.61 (1.21-2.15) 0.001

Diabetes 0=no; 1=yes 1.79 (1.33-2.40) 0.0001

Cigarette smoking 0=no; 1=yes 1.68 (1.26-2.25) 0.0001

Serum uric acid 84 mmol/l (1 SD) 1.15 (1.17-1.50) 0.039

Average Night-time Systolic BP 14 mmHg (1 SD) 1.32 (1.17-1.49) 0.0001

Age 11 years (1 SD) 3.07 (2.65-3.54) 0.0001

Sex 0=women; 1=men 1.66 (1.25-2.19) 0.001

Diabetes 0=no; 1=yes 1.78 (1.33-2.39) 0.0001

Cigarette smoking 0=no; 1=yes 1.73 (1.30-2.30) 0.0001

Serum uric acid 84 mmol/l (1 SD) 1.14 (1.01-1.30) 0.048

_________________________________________________________________________

Abbreviations: SD=Standard deviation; LDL=low density lipoprotein; ECG=electrocardiogram; BP=blood pressure.


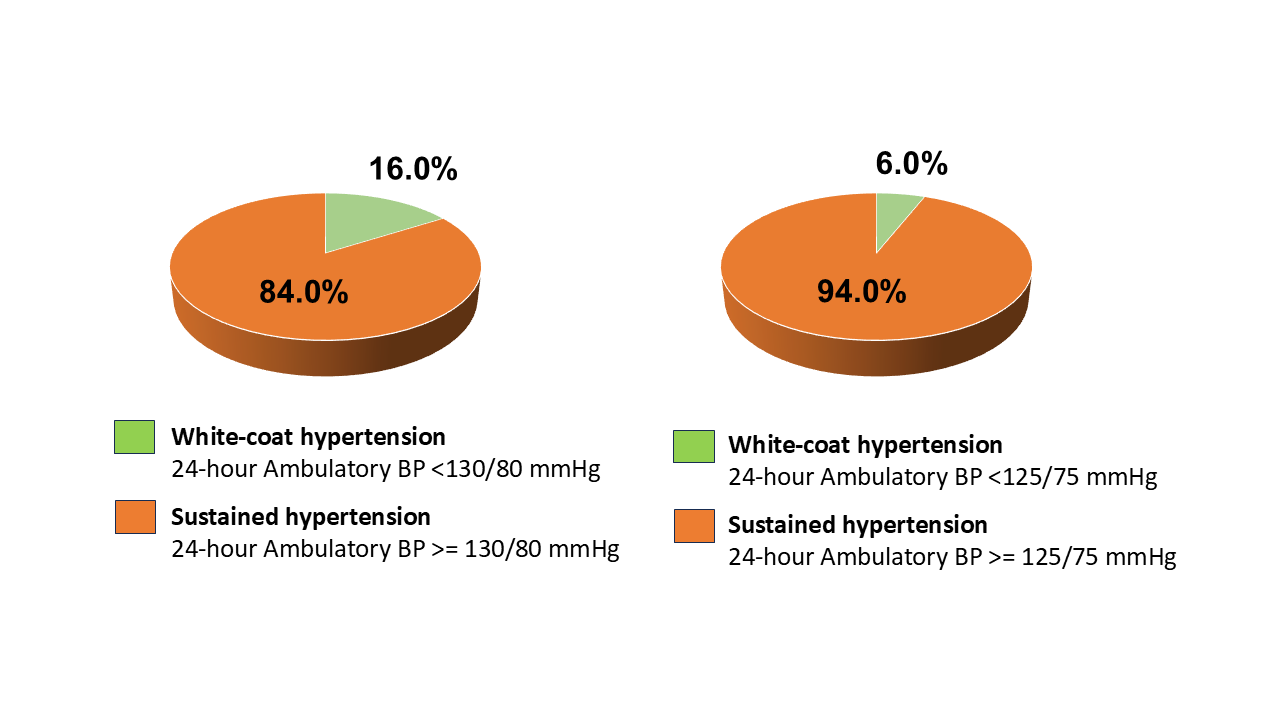


**Figure S1.**

Prevalence of white-coat hypertension using different definitions


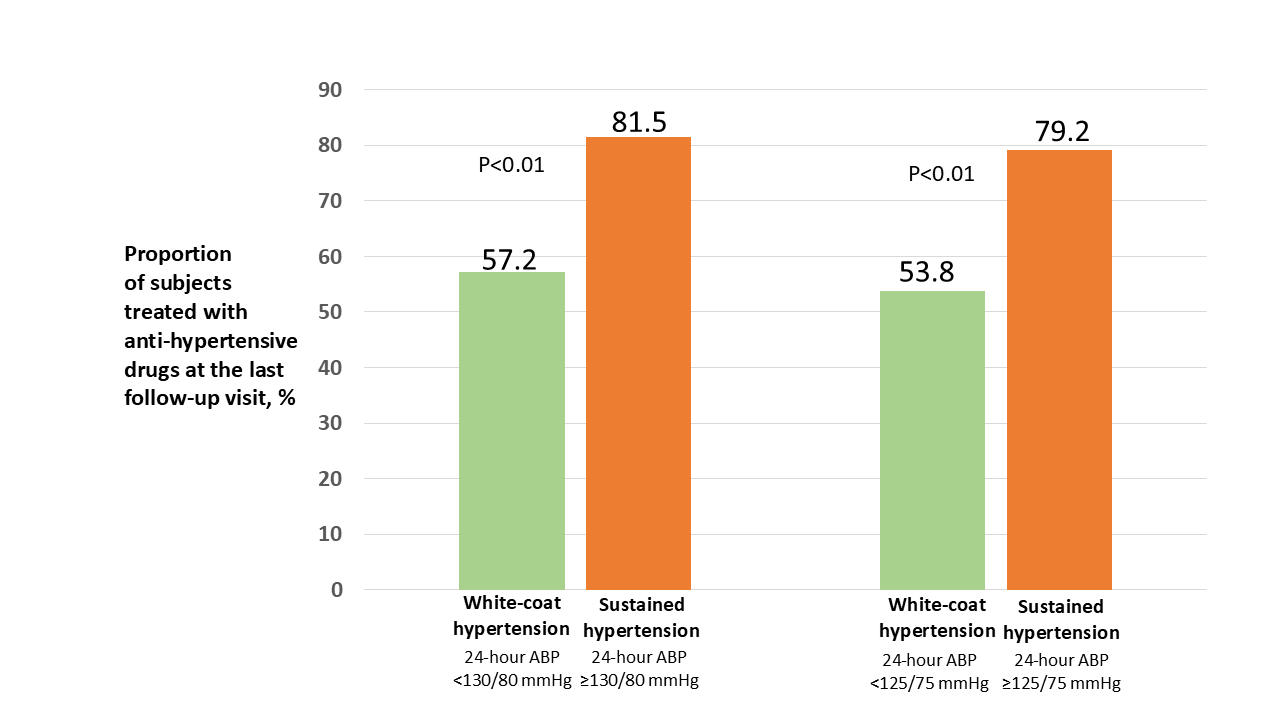


**Figure S2**.

Proportion of subjects receiving antihypertensive drugs at the last follow-up visit.


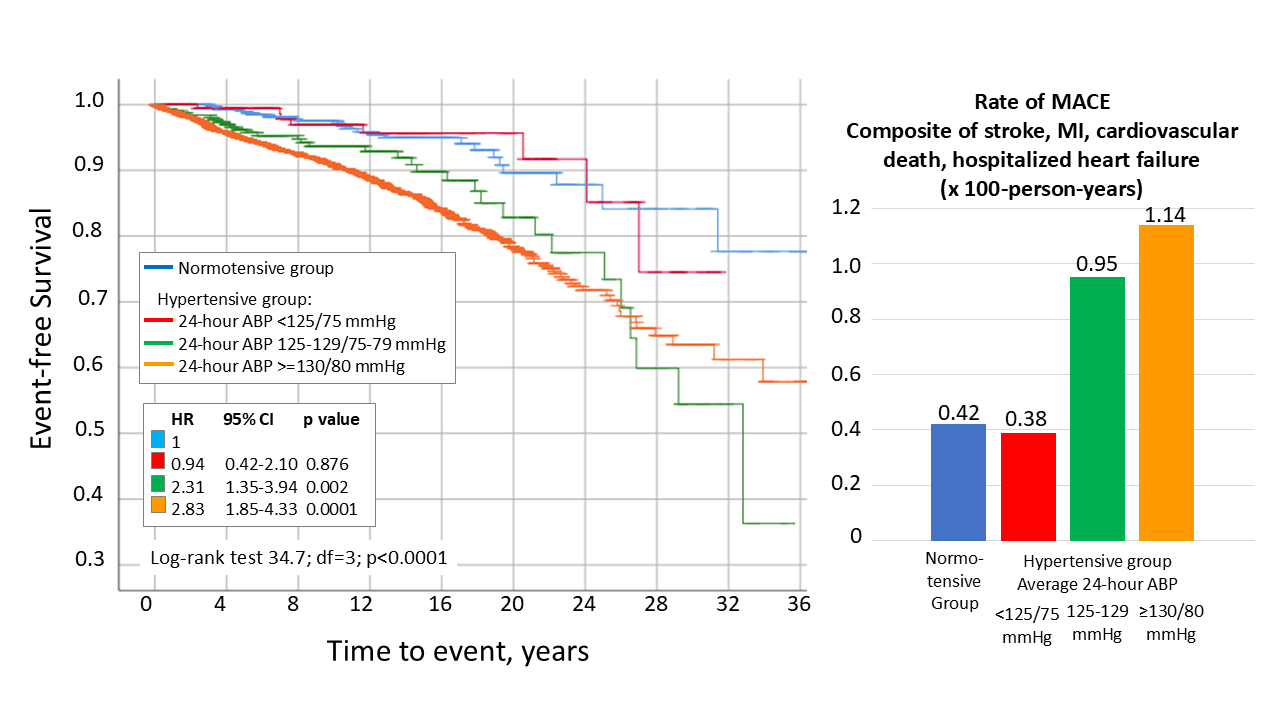


**Figure S3**.

Major adverse cardiovascular events in people with different levels of average 24-hour ambulatory blood pressure used for definition of white-coat hypertension
